# Comparative Analysis of Physicochemical Properties and Biocompatibility of Biomass-Derived and Fossil-Derived Polyvinyl Alcohol Hydrogels: Material Screening for Wound Dressing Applications

**DOI:** 10.3390/gels12010006

**Published:** 2025-12-21

**Authors:** Shanshan Wang, Yun Liu, Han Li, An Xu, Wenqing Liu

**Affiliations:** 1 School of Environmental Science and Optoelectronic Technology, University of Science and Technology of China, Hefei 230026, China; dentistw@mail.ustc.edu.cn (S.W.);; 2 Anhui Province Key Laboratory of Environmental Toxicology and Pollution Control Technology, High Magnetic Field Laboratory, HFIPS, Chinese Academy of Sciences, Hefei 230031, China; 3 Key Laboratory of Environmental Optics and Technology, Anhui Institute of Optics and Fine Mechanics, Hefei Institute of Physical Science, Chinese Academy of Sciences, Hefei 230031, China

**Keywords:** fossil-derived PVA, biomass-derived PVA, hydrogel, physicochemical property, biocompatibility, wound dressing

## Abstract

As one of the most widely used synthetic polymer materials globally, polyvinyl alcohol (PVA) has exhibited promising application potential, especially in the field of wound dressing. Biomass-derived PVA was successfully developed to address the challenges of non-renewable resource depletion and environmental health risks associated with traditional fossil-derived PVA production. However, a knowledge gap still exists regarding the differences between biomass-derived and fossil-derived PVA in terms of their physicochemical and biocompatible properties for wound dressing. This study demonstrated that biomass-derived PVA not only retained the favorable biosafety of conventional PVA (exhibiting no cytotoxicity across multiple cell lines and no induction of inflammatory factors), but also exhibited superior physicochemical properties essential for wound dressing without adding other chemical reagents. Specifically, the light transmittance of biomass-derived PVA hydrogel (>85%) significantly exceeded that of fossil-derived counterparts, highlighting its advantage for wound dressing. Furthermore, the adhesion force of biomass-derived PVA hydrogel to porcine skin was approximately four times that of fossil-derived PVA hydrogel, and the biomass-derived hydrogel exhibited superior drug-loading capacity and more efficient sustained drug release. These findings strongly validated the benefits and applicability of biomass-derived PVA in wound dressing, especially for addressing complex wounds necessitating both physical defense and drug-based intervention.

## 1. Introduction

Polyvinyl alcohol (PVA) is a water-soluble polymer distinguished by its remarkable biocompatibility, biodegradability, mechanical stability and film-forming ability. Owing to these outstanding properties, PVA has been utilized in various fields, including construction, textiles, adhesives, paper manufacturing, cosmetics, food processing, as well as in the medical and hygiene industries [1]. The global PVA market reached a value of USD 1.2 billion in 2024 [2]. Currently, the predominant production routes for fossil-derived PVA can be categorized into two main processes: the ethylene process and the acetylene process [3]. The technological sequence of the ethylene process is as follows: first, ethylene is produced by naphtha cracking; subsequently, vinyl acetate monomer (VAM) is synthesized through catalytic oxidation using ethylene and acetic acid as raw materials, followed by separation and purification steps (with detailed process of the production route was provided in Figure S1 of the Supplementary Materials). The acetylene process can be further divided into two variants: the calcium carbide-based acetylene process (Figure S2) and the natural gas-based acetylene process (Figure S3). The former uses calcium carbide as the raw material to generate acetylene via processes such as high temperature, electric arc, and hydrolysis, while the latter produces acetylene through methane cracking. Both processes utilize acetylene and acetic acid to react under the influence of a zinc acetate catalyst supported on activated carbon to form VAM. After the addition of methanol and a catalyst, VAM undergoes polymerization to generate polyvinyl acetate (PVAc). PVAc then undergoes alcoholysis (saponification) in reaction with sodium hydroxide, ultimately yielding polyvinyl alcohol. However, the fossil-derived production systems are currently confronted with dual crises: first, their operational processes generate significant amounts of various pollutants, including exhaust gases such as NOx, particulate matter, CO, SO_2_, and CO_2_, wastewater discharge from boilers, and solid waste discharge such as tar residue [4,5,6]; second, the unsustainability of petroleum resources and the persistent fluctuations in raw material costs both constrain the future development of the PVA industry. Therefore, researchers tend to develop novel approaches for PVA synthesis using biomass raw materials, such as tubers and sugar cane. Specifically, molasses derived from biomass feedstocks such as sugarcane and tubers has been converted into ethanol through fermentation. This ethanol then underwent a series of sequential process steps including evaporation, catalytic reaction, compression, alkaline washing, drying, and rectification to produce biomass-derived ethylene. The method for synthesizing PVA from this biomass-derived ethylene was consistent with the fossil-derived PVA production process described previously (with detailed synthesis route provided in Figure S4 of the Supplementary Materials). This innovation achieves dual optimization of production sustainability and environmental friendliness [3]. Based on the characteristics of the production processes for fossil-derived PVA and biomass-derived PVA, it could be inferred that both types of PVA products contained residual organic substances (such as acids, esters, aldehydes, and methanol) and metal impurities (such as aluminum, calcium, zinc, and sodium). However, few studies have systematically compared the differences in content regarding these impurities and residues between fossil-derived PVA and biomass-derived PVA. Furthermore, compared to fossil-derived PVA, the physicochemical properties, biocompatibility, and favorable application directions of biomass-derived PVA synthesized via new methods remain unclear and urgently need to be further investigated.

Among the diverse applications of PVA, its development as a hydrogel wound dressing substrate has garnered significant attention [7,8]. Compared with traditional wound dressings, hydrogel dressing demonstrates remarkable advantages, including effective exudate absorption, wound protection, enhanced comfort, and a porous structure that facilitates controlled drug release. PVA has been widely used in hydrogel wound dressing due to its excellent mechanical stability, biocompatibility, and favorable film-forming properties [9]. Furthermore, PVA exhibits unique physical crosslinking properties, which enable PVA to autonomously self-assemble through physical freeze–thaw cycling, forming three-dimensional hydrogel networks without the need for adding conventional chemical crosslinkers (such as glutaraldehyde [10], formaldehyde [11] and epichlorohydrin [12]), thereby eliminating their associated biocompatibility concerns.

Extensive studies have explored the synergistic effects of PVA composites and other polymers in wound dressing applications, such as chitosan [13,14], alginate [15], bacterial cellulose [16], gelatin [17], hyaluronic acid [18], starch [19], poly(N-vinylpyrrolidone) [20], polyethylene glycol [21], polyacrylic acid [22], and polycaprolactone [23]. However, few studies have prepared hydrogel wound dressing using PVA alone as the sole component, primarily attributed to the following reasons. Firstly, PVA hydrogel exhibits inadequate optical transparency, which significantly affects the real-time observation of the wound. Researchers have endeavored to improve the light transmittance of PVA hydrogel via adding other chemical agents (e.g., dimethyl sulfoxide) [24]. However, the incorporation of other substances may introduce potential biological toxicity. Secondly, the poor adhesion of PVA hydrogel often causes detachment from wound site, limiting its clinical utility. Although researchers have attempted to enhance PVA’s mechanical properties by incorporating nanofillers such as carbon nanotubes, graphene oxide, and bacterial cellulose [25,26,27], these modifications inevitably increase fabrication complexity and production costs. Thirdly, conventional freeze–thaw crosslinked pure PVA hydrogel always exhibits an extremely smooth, uniform, and nonporous surface morphology, whose structure significantly compromises the liquid absorption, gas exchange, as well as drug loading and sustained-release capabilities of PVA hydrogel [28,29]. Thus, exploring the feasibility and advantages of biomass-derived PVA as the sole material in hydrogel wound dressing is essential for expanding the application of PVA in wound dressing.

The primary objective of the current study was to (i) systematically compare the physicochemical properties and biocompatibilities of fossil-derived and biomass-derived PVA raw materials, and (ii) uncover the feasibility and advantages of biomass-derived PVA in fabricating multi-functional hydrogel wound dressing. These findings will provide new clues and a theoretical basis for the application of biomass-derived PVA, particularly in the field of biomedical wound dressing.

## 2. Results and Discussion

### 2.1. Physicochemical Characterization of Fossil-Derived and Biomass-Derived PVA and Their Hydrogels

**Figure 1:**
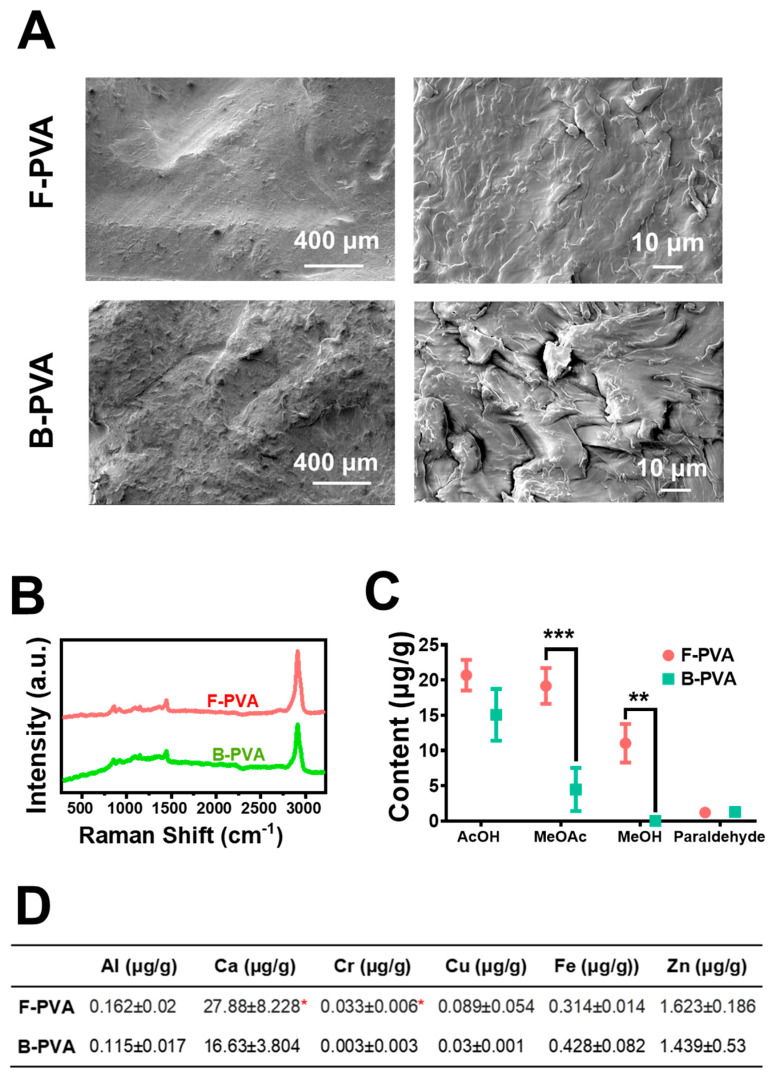
Characterization of fossil-derived and biomass-derived PVA raw materials. (**A**) Representative morphology images of both types of PVA materials observed by Scanning Electron Microscopy (SEM). (**B**) Raman spectra of two types of PVA. (**C**) Organic and (**D**) metal impurity residues of both types of PVA. F-PVA indicated for fossil-derived PVA, B-PVA indicated for biomass-derived PVA. *: *p* < 0.05, **: *p* < 0.01, ***: *p* < 0.001.

The representative morphologies of fossil-derived and biomass-derived PVA materials were investigated via Scanning Electron Microscopy (SEM). As shown in Figure 1A, both types of PVA materials were semi-crystalline polymers. Due to the presence of hydroxyl groups in PVA, they exhibited rough and dispersed surfaces with multiple crystalline domains, which was consistent with the literature [30,31]. Notably, the surface of biomass-derived PVA is marginally rougher than that of fossil-derived PVA. Raman spectra (Figure 1B) of both fossil-derived and biomass-derived PVA exhibited characteristic CH bending and OH bending peaks at both 1445 cm^−1^ and 1376 cm^−1^, a C-C and C-O stretching peak at 1146 cm^−1^, a C-O stretching and OH bending peak at 1097 cm^−1^, a C-C stretching peak at 918 cm^−1^, and a C-C-O stretching peak at 857 cm^−1^, which were consistent with documented PVA vibrational signatures [32]. The organic impurities in both types of PVA materials were quantitatively analyzed using Gas Chromatography (GC) (Figure 1C). Fossil-derived PVA contained higher levels of acetic acid (AcOH), acetic acid methyl ester (MeOAc), and methanol (MeOH) compared to biomass-derived PVA. For instance, the content of MeOAc in fossil-derived PVA was approximately 3 times higher than this in biomass-derived PVA. The content of MeOH in fossil-derived PVA was 11.02 ± 2.74 μg/g, whereas the MeOH content in biomass-derived PVA was below the detection limit of the instrument. Inductively Coupled Plasma-Mass Spectrometry (ICP-MS) analysis (Figure 1D) revealed that the residual concentrations of Al, Ca, Cr, Cu, and Zn in biomass-derived PVA were consistently lower than those in fossil-derived PVA. These results demonstrated that biomass-derived PVA exhibited significantly lower levels of toxic impurities compared to fossil-derived PVA, suggesting superior purity and enhanced biosafety for biomedical applications.

**Figure 2:**
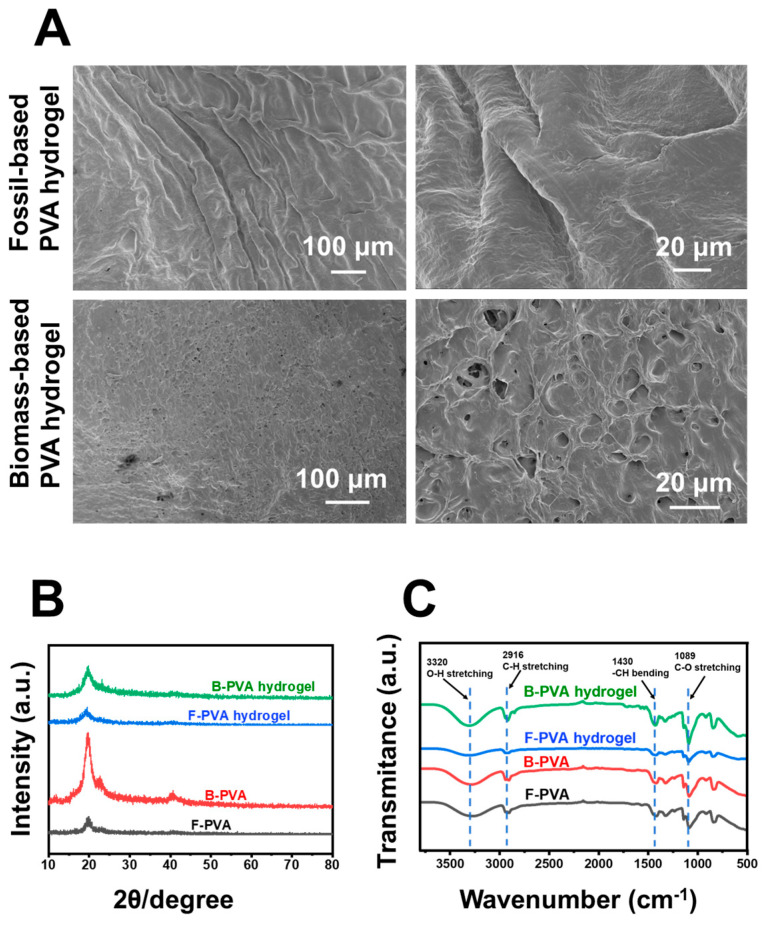
Characterization of fossil-derived and biomass-derived PVA hydrogels. (**A**) Representative morphology images of PVA hydrogels observed by SEM. (**B**) XRD spectra and (**C**) FTIR spectra of PVA and PVA hydrogels. F-PVA indicated for fossil-derived PVA, B-PVA indicated for biomass-derived PVA.

Due to the unique three-dimensional network structure and biomimetic properties, hydrogel preparation for biomedical dressings represents for one of the most important applications of PVA [33]. We thus prepared fossil-derived and biomass-derived PVA hydrogels via cyclic freeze–thaw treatment, and comparatively characterized the physicochemical properties of both types of PVA hydrogels. As shown in Figure 2A, SEM analysis revealed significant differences in the microstructure of fossil-derived and biomass-derived PVA hydrogels. The fossil-derived hydrogel exhibited a dense, non-porous surface morphology, which could be attributed to the tightly packed molecular chains, and was consistent with literature reports [28,29]. In contrast, the biomass-derived PVA hydrogel displayed a highly interconnected 3D porous network structure with an average pore size of approximately 3 to 8 μm. Higher crosslinking density results in a denser hydrogel structure [34]; conversely, it can be inferred that fossil-derived PVA exhibited a higher crosslinking density than biomass-derived PVA. The porous structure of the biomass-derived PVA hydrogel not only enhanced the adhesion, proliferation, and migration abilities of fibroblasts, but also promoted the exchange of trace nutrients, the loading of drugs, and the absorption of wound exudate [35,36]. The distinct microstructures observed through SEM indicated that biomass-derived PVA exhibited greater application potential in the field of wound dressing. The X-ray Diffraction (XRD) analysis (Figure 2B) confirmed that both types of PVA and their corresponding hydrogels exhibited an identical characteristic peak at 19.8°, which was consistent with the characteristic peak of PVA reported in previous literature [37]. The calculated crystallinity of biomass-derived PVA was found to be 14.53%, which was slightly higher than that of fossil-derived PVA (12.94%); correspondingly, the crystallinity of biomass-derived PVA hydrogel was 13.18%, also slightly higher than that of fossil-derived PVA hydrogel (10.52%). These findings could explain the results presented in Figure 1A, indicating that the surface of biomass-derived PVA was slightly rougher than that of fossil-derived PVA. Additionally, analysis results revealed that the crystallite sizes of fossil-derived PVA (4.4 nm) and its hydrogel (4.6 nm) were both larger than those of biomass-derived PVA (3.2 nm) and its hydrogel (3.1 nm). The increase in crystal size leads to a higher entanglement density [38,39] between molecular chains, which explains why the fossil-derived PVA hydrogel in Figure 2A exhibits a more compact structure, while the biomass-derived counterpart is more porous. Fourier Transform Infrared Spectroscopy (FTIR) analysis (Figure 2C) further showed that fossil-derived and biomass-derived PVA materials, along with their corresponding hydrogels, displayed identical characteristic peaks positions. This indicated that the types of functional groups in the PVA hydrogels from both sources did not undergo significant changes before and after successful synthesis via the freeze–thaw process. Notably, the relative peak intensity of the biomass-derived PVA hydrogel at 3320 cm^−1^ was higher than that of the fossil-derived sample, which might imply a greater number of hydroxyl groups contained therein.

### 2.2. Biocompatibility of Fossil-Derived and Biomass-Derived PVA and Their Hydrogels

**Figure 3:**
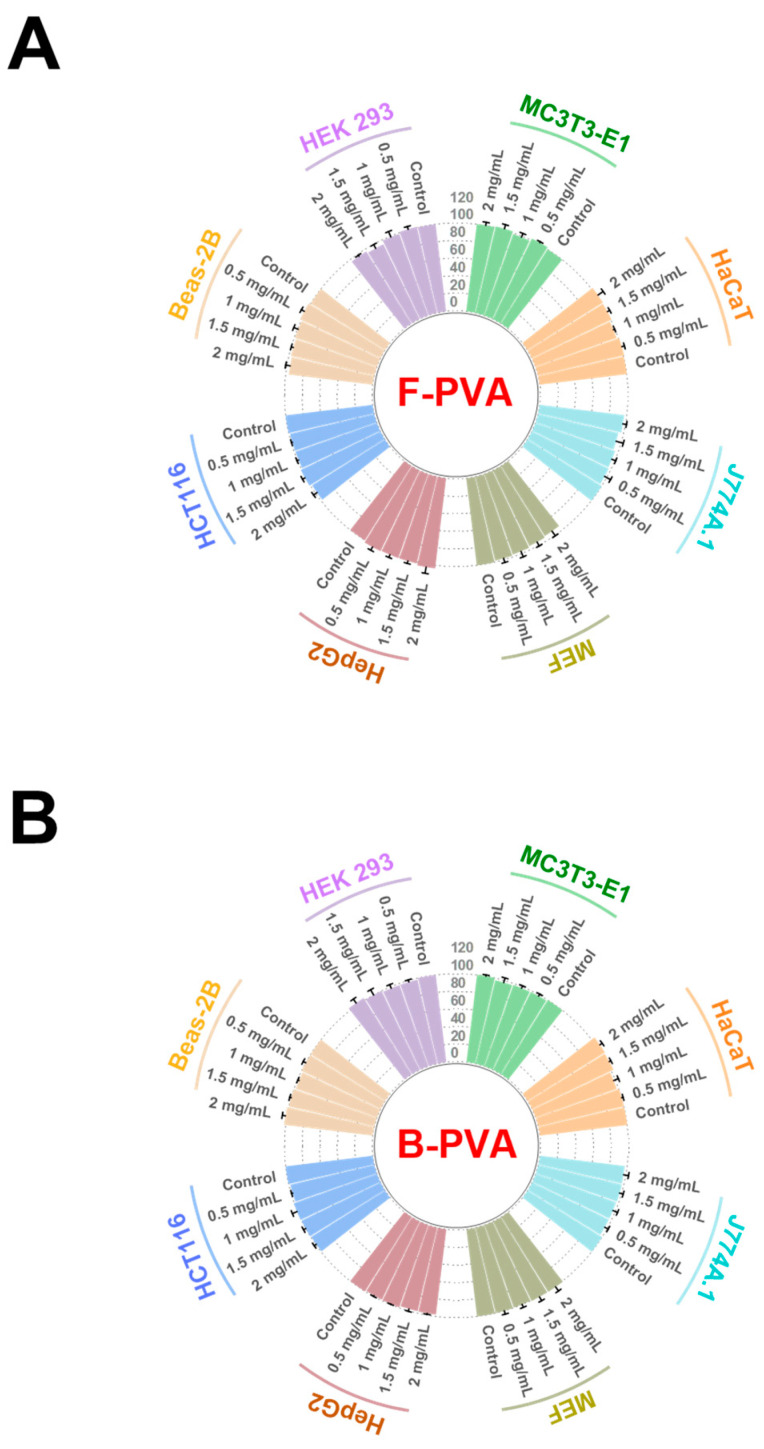
Cytotoxic effects of fossil-derived and biomass-derived PVA on diverse cell lines, including HaCaT, MEF, HCT116, HepG2, Beas-2B, HEK293, J774A.1 and MC3T3-E1. (**A**) The cytotoxicity of fossil-derived PVA. (**B**) The cytotoxicity of biomass-derived PVA. F-PVA indicated for fossil-derived PVA, B-PVA indicated for biomass-derived PVA.

Favorable biocompatibility serves as a fundamental prerequisite for the application of polymeric materials in biomedical fields. Therefore, human epidermal keratinocyte cells (HaCaT), mouse embryonic fibroblast cells (MEF), human colon cancer cells (HCT116), human hepatocellular carcinoma cells (HepG2), human bronchial epithelial cells (Beas-2B), human embryonic kidney cells (HEK293), J774A.1 macrophages (J774A.1), and MC3T3-E1 pre-osteoblastic cells (MC3T3-E1) originated from various important organs, were employed to evaluate the cytotoxicity of fossil-derived and biomass-derived PVA. As shown in Figure 3, the cell viabilities of both fossil-derived and biomass-derived PVA-treated groups exhibited no significant difference compared with the control cells (*p* > 0.05). No cytotoxicity could be observed even when the concentration of PVA was raised to 2 mg/mL, indicating a favorable cell compatibility of both types of PVA materials. As reported by Saif G. Pathan et al. [40], when PVA aqueous solution was diluted with cell culture medium to a final concentration of 75 μM for treating human coronary artery endothelial cells (HCAECs), the addition of PVA did not impair the growth status or viability of the cells. Similarly, diluting PVA to 100 μM in cell culture medium for the treatment of human coronary artery smooth muscle cells (HCASMCs) resulted in favorable growth profiles of HCASMCs without any signs of PVA-induced cytotoxicity, which were consistent with our findings that PVA exhibited favorable biocompatibility. Moreover, our study further confirmed the biocompatibility of PVA by evaluating a broader panel of cell lines, thereby providing more comprehensive evidence for its potential biomedical applications.

**Figure 4:**
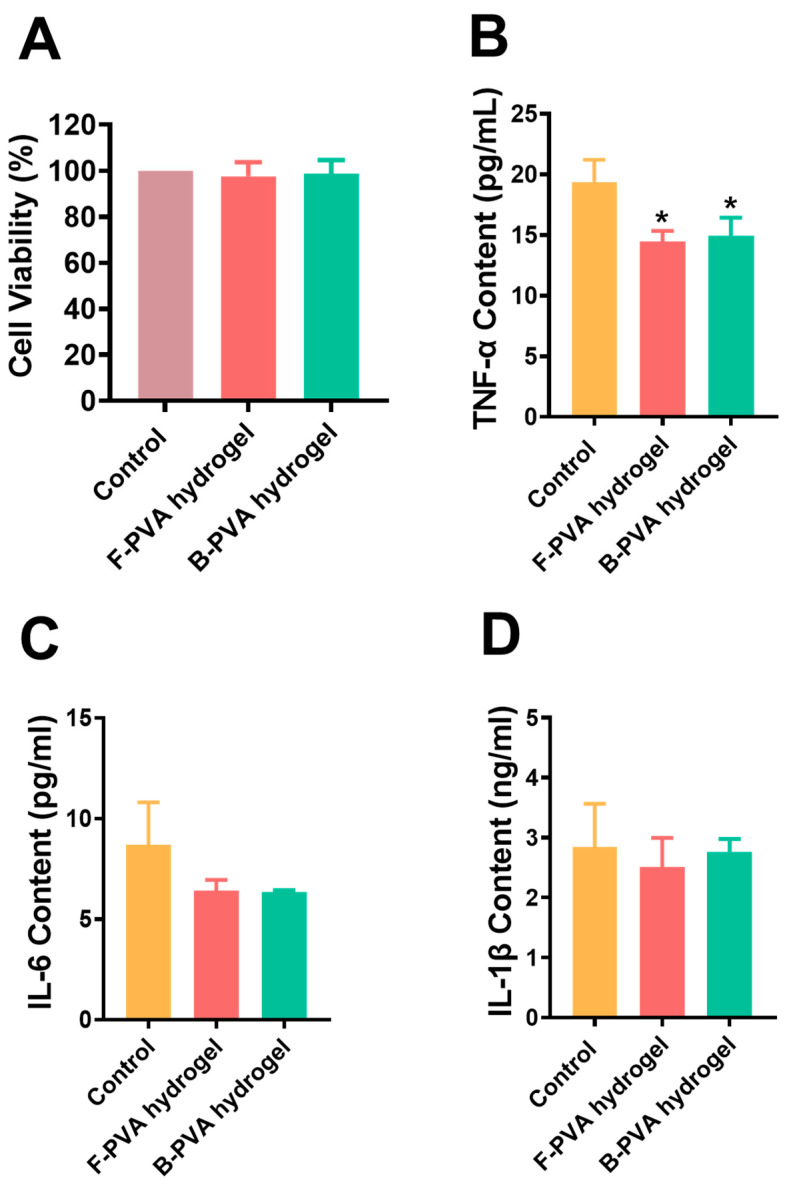
The cytotoxicity and immunotoxicity of fossil-derived and biomass-derived PVA hydrogels. (**A**) The cytotoxicity of fossil-derived and biomass-derived PVA hydrogels on HaCaT cell line. The concentrations of (**B**) TNF-α, (**C**) IL-6, and (**D**) IL-1β in J774A.1 cells after incubated with fossil-derived and biomass-derived PVA hydrogel extracts for 24 h. F-PVA indicated for fossil-derived PVA, B-PVA indicated for biomass-derived PVA. *: *p* < 0.05.

Furthermore, the biocompatibilities of fossil-derived and biomass-derived PVA hydrogels were evaluated through assessing the cytotoxicity and inflammatory response induced by PVA hydrogel extracts. As shown in Figure 4A, neither fossil-derived nor biomass-derived PVA hydrogel extracts exhibited any adverse effects on the viability of HaCaT cells, indicating excellent cytocompatibility for both types of PVA hydrogels. Gun-Woo Oh et al. [41] have demonstrated that PVA hydrogels formed via physical crosslinking (freeze–thaw cycles) exert no adverse effects on cell viability, which was consistent with our findings. In contrast, Mansur, Herman S. et al. [42] reported that chemically crosslinked PVA hydrogels exhibited mild cytotoxicity toward cells, with cell viability occasionally dropping below 80%. These findings collectively demonstrated that PVA hydrogels prepared via physical crosslinking offer significant advantages in terms of cytocompatibility due to the absence of additional crosslinking agents. ELISA results (Figure 4B–D) demonstrated that neither fossil-derived nor biomass-derived hydrogel extracts induced the production of typical pro-inflammatory cytokines (including TNF-α, IL-6, and IL-1β). Instead, a slight decline of the levels of these inflammatory factors could be observed after the addition of PVA hydrogel extracts compared with the control group. Previous studies have reported that PVA microspheres can adsorb inflammatory cytokines in simulated serum [43]. We thus speculated that the PVA hydrogels adsorbed a certain amount of TNF-α, IL-6, and IL-1β, leading to lower inflammatory cytokine levels in the experimental groups compared to the blank control. It is well known that excessive accumulation of pro-inflammatory factors such as TNF-α, IL-6, and IL-1β at wound sites can impair the healing process. The PVA hydrogels, with their three-dimensional network structure, may adsorb these inflammatory cytokines, thereby creating a more favorable microenvironment for wound healing. These findings collectively confirmed the excellent biocompatibility of both types of PVA raw materials and PVA hydrogels, which highlighted their significant role in wound repair applications.

### 2.3. Optical Transmittance, Swelling and Adhesion Capacities of Fossil-Derived and Biomass-Derived PVA Hydrogels

**Figure 5:**
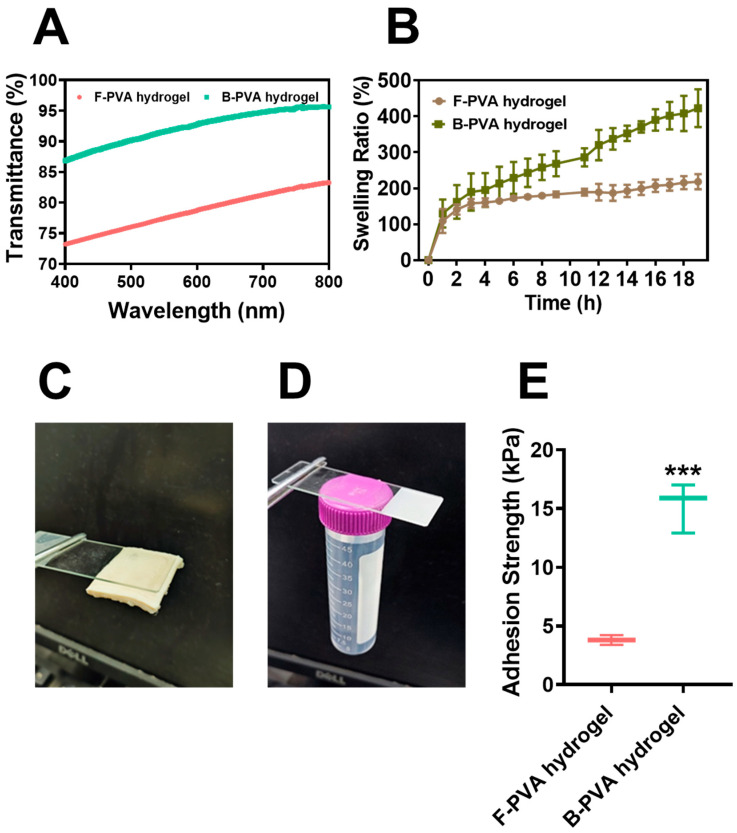
Physical properties of fossil-derived and biomass-derived PVA hydrogels. (**A**) Transmittance of PVA hydrogels. (**B**) The water absorption and swelling capacity of PVA hydrogels. Photographs showing the adhesion force between biomass-derived PVA hydrogels and (**C**) porcine dermal tissue or (**D**) EP tube filled with 50 mL water. (**E**) The adhesion strength between porcine dermal tissue and the two types of PVA hydrogels. F-PVA indicated for fossil-derived PVA, B-PVA indicated for biomass-derived PVA. ***: *p* < 0.001.

Transparency is a critical characteristic of wound dressing, particularly for wounds requiring close monitoring. Transparent dressings enable direct visualization of wound changes through the material, eliminating the need for frequent removal and thus minimizing secondary trauma, which significantly enhances clinical care efficiency [44]. As illustrated in Figure 5A, under identical freeze–thaw cycling conditions, the biomass-derived PVA hydrogels demonstrated outstanding optical transmittance, maintaining over 85% transparency throughout the visible spectrum. This performance significantly surpassed that of fossil-derived PVA hydrogels, whose transmittance was below 85% and consistent with previous reports in the literature [24]. The biomass-derived PVA hydrogel prepared by a simple, safe, and environmentally friendly method exhibited a transparency of 87.60 ± 4.45% at 600 nm, which was higher than that of many transparent hydrogels fabricated via complex processes reported previously [45,46]. The light transmittance of PVA hydrogels is closely related to their crosslinking density, crystallinity [24], and crystal size [47]. The SEM characterization results in Figure 2A showed that the fossil-derived PVA hydrogel had a denser structure, which contributed to its inferior light transmittance as compared to that of the biomass-derived PVA hydrogel. In addition, the XRD analysis results in Figure 2B indicated that the two hydrogels exhibited a slight difference in crystallinity, while the smaller crystal size of the biomass-derived PVA hydrogel might have been another key factor enhancing its superior light transmittance. The high transparency of biomass-derived PVA hydrogels endowed them with great potential in biomedical applications, such as transparent wound dressings and functional contact lenses.

Swelling capacity, a crucial indicator for evaluating the crosslinking density and internal structure of hydrogels, quantitatively reflects the water absorption potential within polymer networks [48,49]. As indicated in Figure 5B, the biomass-derived PVA hydrogels demonstrated significantly superior swelling capability compared to their fossil-derived counterparts. Moreover, the discrepancy in swelling ratio between biomass-derived PVA hydrogels and fossil-derived PVA hydrogels grew increasingly pronounced as time progressed. The equilibrium swelling ratio of biomass-derived PVA hydrogels was approximately twice that of fossil-derived PVA hydrogels. This enhanced water absorption capacity of biomass-derived PVA hydrogels could be attributed to their well-developed three-dimensional network structure and higher porosity (corresponding to the SEM results shown in Figure 2A). From the perspective of clinical application, such excellent swelling properties make biomass-derived PVA hydrogels particularly suitable for wound dressing applications, where they can effectively absorb wound exudates, facilitate gas exchange and maintain optimal moisture levels at the wound site [48,50].

Apart from outstanding optical transmittance and swelling capacity, the appropriate interface adhesion ability is also a key functional parameter of wound dressing hydrogel, which directly determines the mechanical coupling effect at the tissue interface, the therapeutic durability, and the comfort level. In this study, the PVA hydrogel derived from biomass not only formed a stable adhesion interface on porcine dermal tissue (Figure 5C), but also firmly adhered to an EP tube containing 50 mL of solution without falling off (Figure 5D). Quantitative analysis revealed that the adhesion strength between the biomass-derived PVA hydrogel and porcine skin could reach to 15.27 ± 2.122 kilopascals, which was approximately four times that of fossil-derived PVA hydrogel (*p* < 0.05) (Figure 5E). The difference in adhesion strength between the two PVA hydrogels could be attributed to two aspects: on the one hand, it was related to the differences in their surface chemical properties—as shown in the FTIR spectroscopy results in Figure 2C, the biomass-derived PVA hydrogel had a higher relative content of hydroxyl groups than the fossil-derived PVA hydrogel, and the additional hydroxyl groups could form hydrogen bonds on the material surface, thereby enhancing adhesion performance [51]. On the other hand, previous literature had reported that the adhesion of PVA hydrogels decreased significantly with increasing crosslinking density [52]. The SEM results in Figure 2A demonstrated that, compared with the fossil-derived PVA hydrogel, the biomass-derived PVA hydrogel possessed a well-developed network structure on its surface and a relatively lower crosslinking density, which also contributed to its superior adhesion strength. Compared to traditional fossil-derived PVA hydrogels (with inadequate adhesion [25,26,27]) and high-adhesion polymers (e.g., chitosan [53,54]), this moderate and suitable adhesion force of the biomass-derived PVA hydrogel ensures that wound dressings remain firmly adhered to the skin surface while minimizing discomfort during dressing change. Collectively, all the tested physical properties indicated that biomass-derived PVA hydrogels were more suitable for preparing wound dressings compared to fossil-derived PVA hydrogels.

### 2.4. Drug Loading and Controlled Release Performance of Fossil-Derived and Biomass-Derived PVA Hydrogels

**Figure 6:**
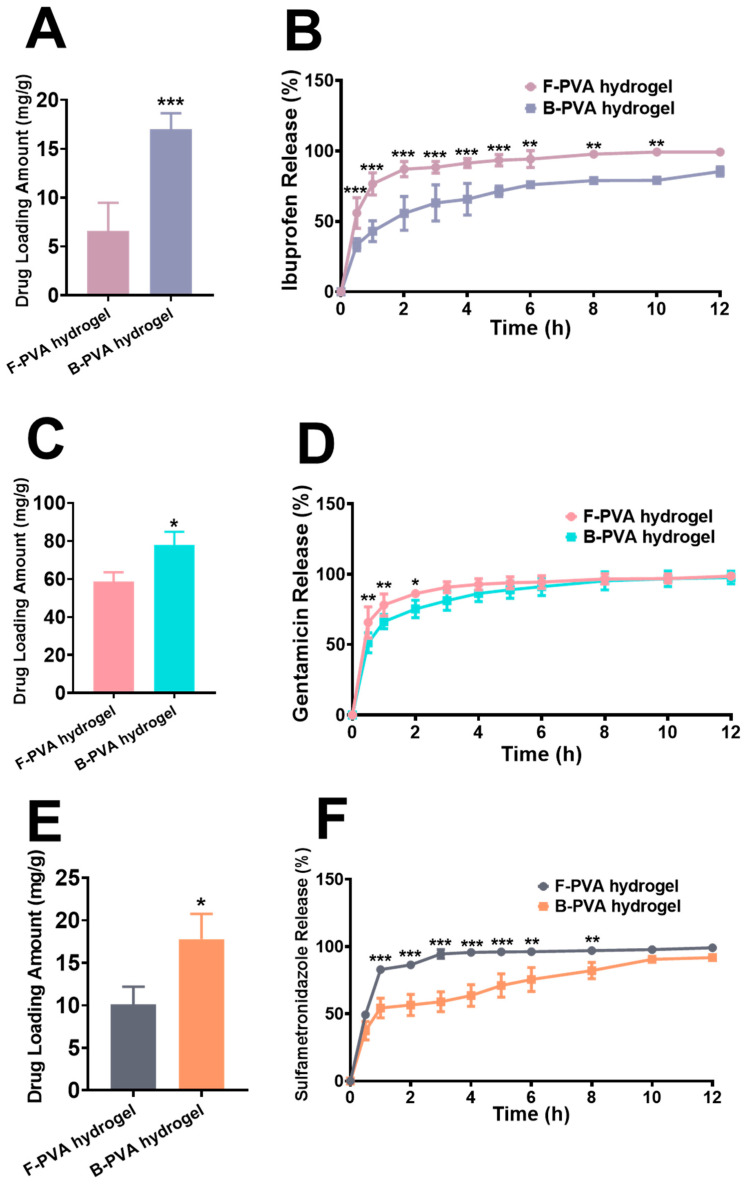
Drug loading capacities and sustained-release profiles of fossil-derived and biomass-derived PVA hydrogels. Drug loading capacities of (**A**) ibuprofen, (**C**) gentamicin and (**E**) sulfamethoxazole of both types of PVA hydrogels. Sustained-release profiles of (**B**) ibuprofen, (**D**) gentamicin and (**F**) sulfamethoxazole of both types of PVA hydrogels. F-PVA indicated for fossil-derived PVA, B-PVA indicated for biomass-derived PVA. *: *p* < 0.05, **: *p* < 0.01, ***: *p* < 0.001.

For complex wounds such as infected or severely painful cases, adjunctive pharmacotherapy becomes necessary, thus requiring an ideal hydrogel dressing carrier to possess both drug-loading and sustained-release capabilities. Given that ibuprofen is commonly used for pain management, while gentamicin and sulfamethoxazole are standard antibiotics for infection control, this study systematically investigated the drug-loading efficiencies and controlled release profiles of these three therapeutic agents in both types of PVA hydrogels. Compared to fossil-derived PVA hydrogels, biomass-derived PVA hydrogels achieved significantly higher drug loading efficiencies for ibuprofen (16.99 ± 1.649 mg/g) (Figure 6A), gentamicin (77.86 ± 6.908 mg/g) (Figure 6C), and sulfamethoxazole (17.79 ± 2.960 mg/g) (Figure 6E), with their drug loading amounts being approximately 2.57, 1.33, and 1.76 times those of fossil-derived PVA hydrogels, respectively. The drug release experiments (Figure 6B,D,F) further demonstrated that the biomass-derived PVA hydrogels also exhibited superior sustained-release performance compared to their fossil-derived counterparts. In general, when the three drugs (ibuprofen, gentamicin sulfate, and sulfamethoxazole) were loaded into the two types of carriers respectively, the release rate in the biomass-derived PVA hydrogels was significantly slower than that in the fossil-derived PVA hydrogels. Taking sulfamethoxazole as an example, the fossil-derived PVA hydrogel exhibited a significant drug burst release phenomenon: the amount of drug released reached 50% of the total amount within 30 min after the start of release; the drug release curve basically tended to be flat after approximately 3 h, indicating that most of the drug had been released just within a short time. In contrast, the biomass-derived PVA hydrogel had a milder initial release rate, with the drug release amount within 30 min being less than 50%; in the time period of 6 to 12 h, a continuous drug release process could still be observed. Similar to our work, Desheng Liu et al. [55] developed a highly transparent PVA hydrogel via desalination followed by dialysis, and they physically loaded pranoprofen into the hydrogel by immersing it in a pranoprofen solution. However, the cumulative release of pranoprofen attained saturation in only 60 min. This comparison has further confirmed the application advantages of transparent biomass-derived PVA hydrogels.

Biomass-derived PVA hydrogels exhibited better drug loading capacity and controlled release performance compared to fossil-derived ones, mainly due to their unique porous network structure. Specifically, the 3D network increased the hydrogel’s specific surface area and provided more binding sites for drug molecules, enabling higher drug loading efficacy and more stable sustained release. These experimental data clearly demonstrated that the biomass-derived PVA hydrogels, as a novel wound dressing material, not only retained the inherent advantages of hydrogel dressings, but also exhibited excellent drug carrier functionality. Importantly, this material could be flexibly loaded with various drugs (e.g., antibiotics and non-steroidal anti-inflammatory drugs) according to clinical needs and achieve controlled release, thereby synergistically providing multiple therapeutic effects including anti-infection and anti-inflammatory/analgesic actions. This integrated “dressing-carrier” design may offer another solution for comprehensive treatment of complex wounds, particularly suitable for clinical scenarios requiring simultaneous physical wound protection and pharmacological therapy.

## 3. Conclusions

This study demonstrated that biomass-derived PVA hydrogels, as a more environmentally benign alternative, exhibited superior physicochemical properties for wound dressings compared with traditional fossil-derived PVA hydrogels. Notably, biomass-derived PVA hydrogels not only displayed high biocompatibility, but also achieved sufficient transparency (>85%) through an autogenous gelation mechanism without adding exogenous chemicals, thereby ensuring greater biosafety, convenience, and economy. Furthermore, in comparison with fossil-derived PVA hydrogels, biomass-derived PVA hydrogels exhibited more appropriate adhesion ability to skin tissues (15.27 ± 2.122 kilopascals, approximately four times that of fossil-derived PVA hydrogel), and demonstrated superior performance in both drug loading and sustained drug release. Collectively, the design of biomass-derived PVA hydrogels provided an innovative, biosafe and convenient strategy for developing multi-functional wound-repair dressings encompassing “transparency-visualization, mild adhesion, and intelligent controlled-release”, especially for treating complex wounds requiring both physical wound protection and pharmacological therapy.

## 4. Materials and Methods

### 4.1. Materials

Both fossil-derived PVA and biomass-derived PVA, specified as PVA-2699 (wherein the degree of polymerization (DP) is 2600 and the degree of alcoholysis (DA) is 99%), were obtained from Anhui Wanwei Group Co., Ltd. (Chaohu, China). The Cell Counting Kit-8 (CCK-8) was purchased from Beyotime Biotechnology (Shanghai, China). The ELISA kits were purchased from Cusabio Technology LLC (Wuhan, China). Ibuprofen (≥98% purity) and Sulfamethoxazole (≥98% purity) were purchased from Sigma-Aldrich Co. LLC (St. Louis, MO, USA). Gentamicin sulfate (≥98% purity) was purchased from Henan Huaxu Biotechnology Co., Ltd. (Zhengzhou, China).

### 4.2. Preparation of PVA Aqueous Solutions and PVA Hydrogels

5 g fossil-derived or biomass-derived PVA was added into 100 mL ultra-pure water, separately. PVA particles were gradually dispersed into ultrapure water under continuous magnetic stirring at 25 ± 1 °C for 30 min to achieve uniform wetting. Then the temperature slowly increased the temperature to 85 °C until the solid material was completely dissolved. The solution was degassed by standing at room temperature, and then poured into molds for cyclic freeze–thaw treatment (−20 °C freezing for 8 h followed by 25 °C thawing for 8 h per cycle). Next, this process was repeated for three times in order to yield PVA hydrogels.

### 4.3. Characterization of Fossil-Derived and Biomass-Derived PVA Materials and Hydrogels

Following gold-sputtering pretreatment on the dried raw materials of biomass-derived PVA and fossil-derived PVA, as well as the two types of freeze-dried PVA hydrogels, their surface morphologies were characterized using a field-emission scanning electron microscope (FE-SEM, Thermo Fisher Scientific, Axia ChemiSEM, Waltham, MA, USA) at an accelerating voltage of 5 kV.

Raman Spectroscopy measurement of PVA materials were conducted on a confocal Raman Spectroscopy system (Horiba, LabRAM HR Evolution, Longjumeau Cedex, IDF, France), with an excitation wavelength of 532 nm and a spectral range of 300–3200 cm^−1^.

The quantitative analysis of organic impurities in PVA materials was conducted using a Gas Chromatograph (Agilent Technologies, 8860, San Clara, CA, USA). For acetic acid, methyl acetate, and methanol, the column was 0.32 mm × 30 m × 0.25 μm, with carrier gas: N_2_ at 1.0 mL·min^−1^, split ratio 1:1, H_2_ flow 30 mL·min^−1^, air flow 300 mL·min^−1^, detector temperature 250 °C, inlet temperature 200 °C, and temperature program: 40 °C (8 min) → 150 °C (10 °C·min^−1^, 2 min); headspace conditions included 80 °C for 30 min equilibration, 0.2 min pressurization, and 2.0 mL injection volume. For paraldehyde, the column was CPWAX 52 CB (50 m × 0.32 mm × 1.0 μm), with carrier gas: high-purity He at 1.0 mL·min^−1^, split ratio 5:1, H_2_ flow 35 mL·min^−1^, air flow 350 mL·min^−1^, detector and inlet temperatures were 250 °C, and temperature program: 52 °C (5 min) → 150 °C (15 °C·min^−1^, 2 min) → 200 °C (60 °C·min^−1^, 5 min); headspace conditions included 125 °C for 45 min equilibration, sample loop temperature 135 °C, transfer line temperature 145 °C, and 1 mL injection volume. The residual metal ions in PVA materials were analyzed by Inductively Coupled Plasma-Mass Spectrometry (Thermo Fisher Scientific, iCAP 7400, Waltham, MA, USA).

X-ray diffraction (XRD) analysis was conducted on two types of PVA materials and the corresponding two types of freeze-dried PVA hydrogels using an X-ray diffractometer (Rigaku, TTR-III, Tokyo, Japan). The test conditions were set as follows: tube voltage 40 kV, tube current 200 mA, scanning range 2θ = 10–90°, step size 0.02°, and scanning rate 15°/min.

Fourier Transform Infrared Spectroscopy (FTIR) analyses of PVA materials and PVA hydrogels were conducted using a Fourier Transform Infrared spectrometer (Thermo Fisher Scientific, Nicolet iS50, Waltham, MA, USA), which accumulated from 128 scans in the 400–4000 cm^−1^ range with a resolution of 4 cm^−1^.

### 4.4. Cell Culture and Exposure

#### 4.4.1. Cell Culture

Cells were all from the Cell Resource Center of Shanghai Institute of Life Sciences, Chinese Academy of Sciences. J774A.1 macrophages (J774A.1) were cultured in high-glucose Dulbecco’s Modified Eagle Medium (DMEM, HyClone, Logan, UT, USA) supplemented with 15% fetal bovine serum (FBS, Excell Bio Co., Ltd., Shanghai, China) and 1% penicillin/streptomycin (Biosharp Life Sciences Co., Ltd., Anhui, China). MC3T3-E1 pre-osteoblastic cells (MC3T3-E1) were maintained in α-MEM medium containing 10% FBS and 1% penicillin-streptomycin. Other cell lines, including human epidermal keratinocyte cells (HaCaT), mouse embryonic fibroblast cells (MEF), human colon cancer cells (HCT116), human hepatocellular carcinoma cells (HepG2), human bronchial epithelial cells (Beas-2B), and human embryonic kidney cells (HEK293), were all cultured in DMEM supplemented with 10% FBS and 1% penicillin-streptomycin. All cells were incubated at 37 °C under 5% CO_2_.

#### 4.4.2. Exposure of Cells to PVA Raw Materials

The PVA aqueous solutions were diluted with culture medium to 0.5, 1, 1.5, and 2 mg/mL, and were then applied to the aforementioned cell lines for 24 h, with the control group receiving PVA-free culture medium.

#### 4.4.3. Exposure of Cells to PVA Hydrogels

According to ISO 10993-12/GB/T 16886.12-2017 [56], the extracts of PVA hydrogels were prepared at the extraction ratio of 1.25 cm^2^/mL hydrogel surface area to culture medium volume at 37 °C. After 24 h of extraction, the leach liquor was then applied to cells for another 24 h, with blank culture medium as the control.

### 4.5. Evaluation of the Cytotoxicity of PVA

Cell viability was assessed using the Cell Counting Kit-8 (CCK-8). Cells were seeded in 96-well plates. Following exposure to different materials, the waste solution was discarded. After washing with PBS, 100 μL of CCK-8 solution (diluted to 10% with basal medium) was added to each well. After incubation at 37 °C for 1–4 h in the dark, the absorbance was measured at 450 nm using a microplate reader (Molecular Devices, SpectraMax^®^ i3x, San Jose, CA, USA).

### 4.6. Inflammatory Cytokines Determination

After exposing J774A.1 cells to PVA hydrogels for 24 h using the aforementioned method, the cell culture supernatants were collected, and the levels of inflammatory cytokines, including TNF-α, IL-6, and IL-1β, were measured using ELISA kits according to the manufacturer’s instructions.

### 4.7. Optical Transparency Measurement

The optical transparency of the hydrogels was quantified using a UV-Vis spectrophotometer. Fossil-derived and biomass-derived PVA hydrogels were sectioned into 0.5 mm-thick rectangular specimens and absorbance measurements were performed at 400–800 nm. Thus, the transmittance of the hydrogels was calculated.

### 4.8. Swelling Capacity Measurement

The freeze-dried PVA hydrogels were weighed and immersed in distilled water to assess their swelling capacity at room temperature. The swollen hydrogels were removed, surface moisture was blotted with filter paper, and they were periodically weighed until reaching swelling equilibrium. The formula for calculating the swelling ratio was as follows:

(1) $$Q=\frac{W2\;-\;W1}{W1}$$

where Q is the equilibrium water absorption ratio (grams of water per gram of sample), and W1 and W2 represent the masses of the hydrogel sample before and after swelling, respectively.

### 4.9. Adhesion Assessment

Hydrogel samples and porcine dermal tissues were bonded to glass substrates. The hydrogel-tissue interface was compressed under 0.98 N normal force for 5 min contact time. Lap-shear adhesion testing was performed on a universal testing machine at 5 mm/min crosshead speed until interfacial failure. Maximum tensile force and displacement were recorded, with adhesion strength calculated as peak force divided by initial contact area.

### 4.10. Drug Loading and Drug Sustained-Release Experiments

Ibuprofen, gentamicin, and sulfamethoxazole were individually dissolved or ultrasonically dispersed in ultrapure water to prepare 2 mg/mL solutions or suspensions. Two types of lyophilized xerogels (0.1 g) were separately immersed in the corresponding drug solutions/suspensions (10 mL) and incubated at room temperature for 72 h to achieve drug loading equilibrium. After immersion, the hydrogels were retrieved, and any surface-adhered drug solution/suspension was blotted dry with filter paper. The drug concentrations in the solutions or suspensions before and after hydrogel immersion were measured using an ultraviolet-visible (UV-Vis) spectrophotometer, and the drug loading capacity was quantified by referencing a pre-established calibration curve. The drug loading capacity of the PVA hydrogels was determined using the following formula:

(2) $$L=\frac{C0V0-C1V1}{M}$$

where L is the loading rate of the drug; C_0_ is the initial drug concentration (mg/mL); C_1_ is the equilibrium drug concentration post-loading (mg/mL); V_0_ is the volume (mL) of the drug solution/suspension before immersion, and V_1_ is that after immersion; M is the mass (g) of the dry hydrogel.

The drug release experiment of the drug-loaded hydrogels was conducted in a container filled with PBS (40 mL) under simulated physiological conditions (37 ± 0.5 °C, 100 rpm shaker). Cumulative drug release was assessed through periodic sampling with iso-volume buffer replenishment at predetermined time intervals. UV-Vis spectrophotometry was employed to quantify drug concentrations against a validated calibration curve. Thus, the cumulative drug release percentage was calculated.

### 4.11. Statistical Analysis

All experiments in this study were independently repeated three or more times. Data were presented as the mean ± SD (standard deviation). Statistical analysis methods mainly included independent samples *t*-test and one-way ANOVA. *p* < 0.05 was considered statistically significant and indicated a significant difference.

## Data Availability

Dataset available on request from the authors (The raw data supporting the conclusions of this article will be made available by the authors on request.

## Supplementary Materials

The following supporting information can be downloaded at: https://www.mdpi.com/article/10.3390/gels12010006/s1, Figure S1: Flow diagram of fossil-derived PVA production via petroleum–ethylene process; Figure S2: Flow diagram of fossil-derived PVA production via calcium carbide–acetylene process; Figure S3: Flow diagram of fossil-derived PVA production via natural gas–acetylene process; Figure S4: Process flow diagram for biomass-derived PVA production.
