# Supplementary material for: Comparative Analysis of Physicochemical Properties and Biocompatibility of Biomass-Derived and Fossil-Derived Polyvinyl Alcohol Hydrogels: Material Screening for Wound Dressing Applications
Source: Gels. 2025 Dec 21;12(1):6. doi: 10.3390/gels12010006 (PMC12841456; doi:10.3390/gels12010006)
Supplement: Supplementary file 1 [file gels-12-00006-s001.zip › gels-3992812-supplementary.pdf]

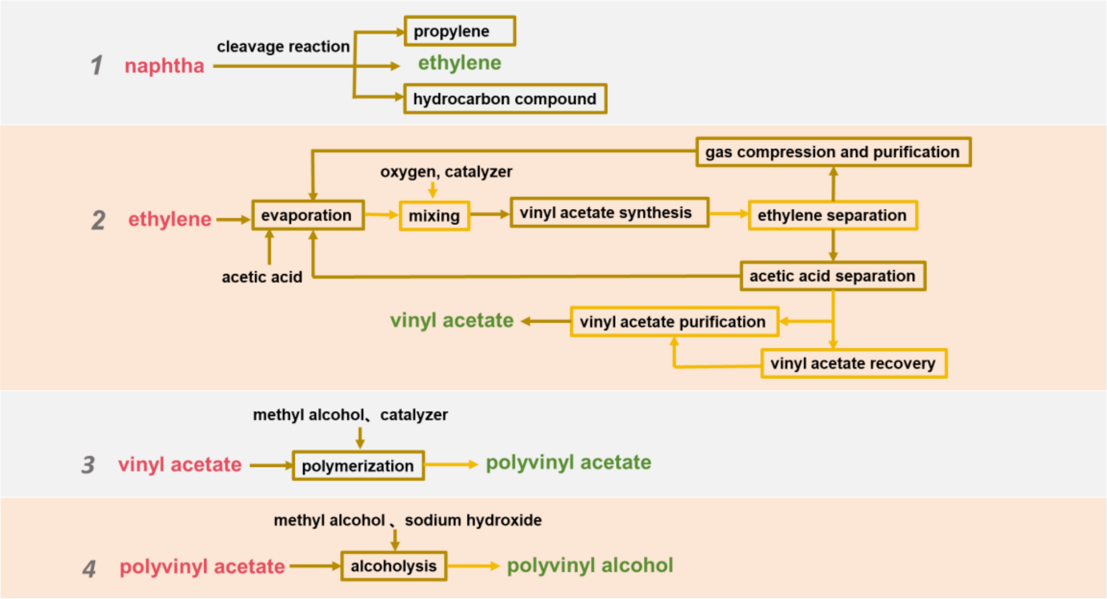

Figure S1. Flow diagram of fossil-derived PVA production via petroleum–ethylene process.

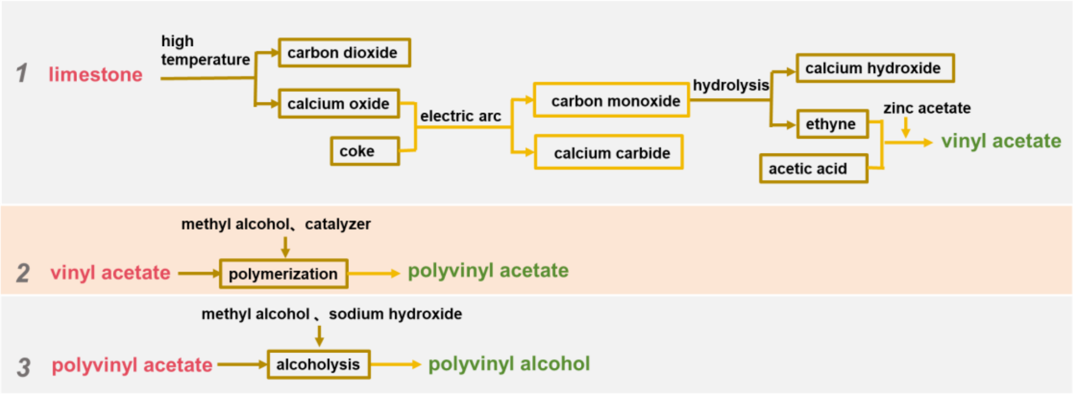

Figure S2. Flow diagram of fossil-derived PVA production via calcium carbide–acetylene process.

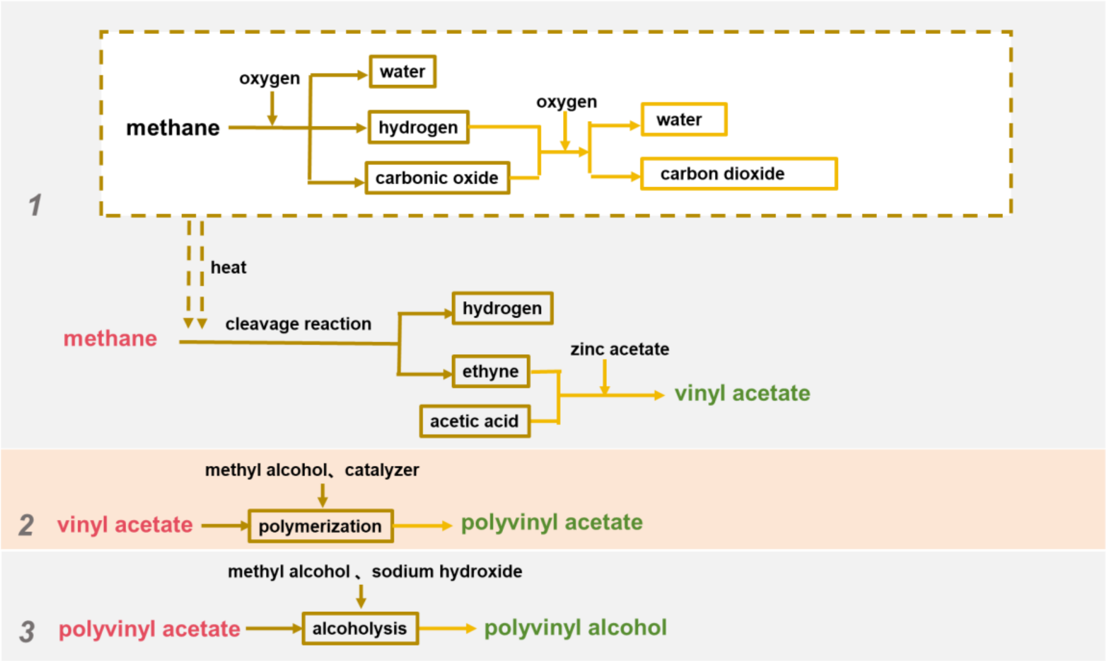

Figure S3. Flow diagram of fossil-derived PVA production via natural gas–acetylene process.

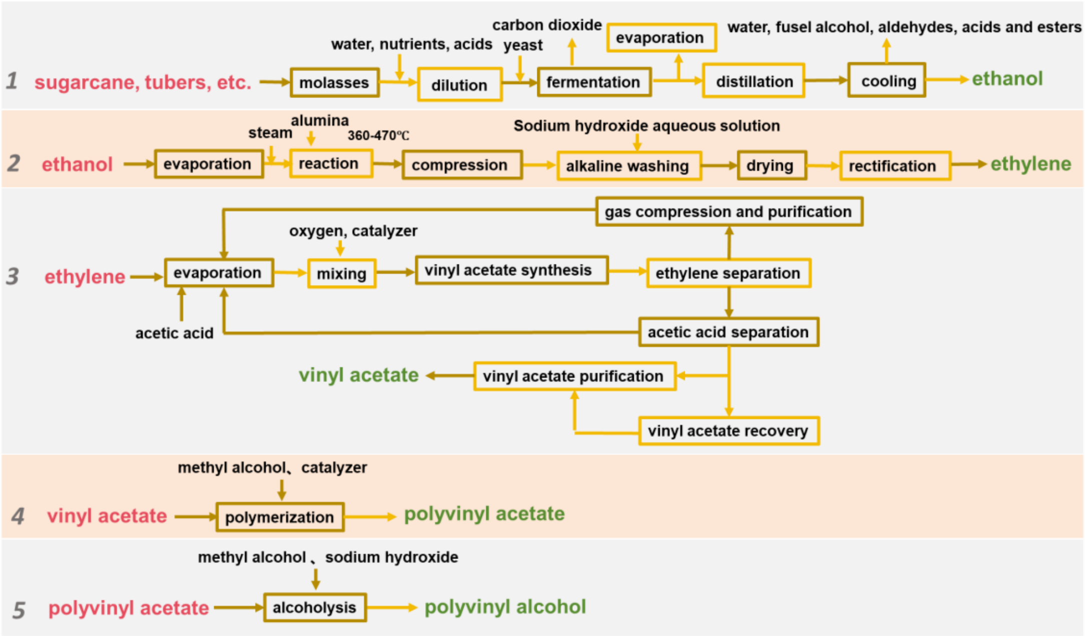

Figure S4. Process flow diagram for biomass-derived PVA production.
